# High-power ultralong-wavelength Tm-doped silica fiber laser cladding-pumped with a random distributed feedback fiber laser

**DOI:** 10.1038/srep30052

**Published:** 2016-07-15

**Authors:** Xiaoxi Jin, Xueyuan Du, Xiong Wang, Pu Zhou, Hanwei Zhang, Xiaolin Wang, Zejin Liu

**Affiliations:** 1 College of Optoelectronic Science and Engineering, National University of Defense Technology, Changsha 410073, China

## Abstract

We demonstrated a high-power ultralong-wavelength Tm-doped silica fiber laser operating at 2153 nm with the output power exceeding 18 W and the slope efficiency of 25.5%. A random distributed feedback fiber laser with the center wavelength of 1173 nm was employed as pump source of Tm-doped fiber laser for the first time. No amplified spontaneous emissions or parasitic oscillations were observed when the maximum output power reached, which indicates that employing 1173 nm random distributed feedback fiber laser as pump laser is a feasible and promising scheme to achieve high-power emission of long-wavelength Tm-doped fiber laser. The output power of this Tm-doped fiber laser could be further improved by optimizing the length of active fiber, reflectivity of FBGs, increasing optical efficiency of pump laser and using better temperature management. We also compared the operation of 2153 nm Tm-doped fiber lasers pumped with 793 nm laser diodes, and the maximum output powers were limited to ~2 W by strong amplified spontaneous emission and parasitic oscillation in the range of 1900–2000 nm.

In recent decade, great progress has been made in fiber lasers operating at ~2 μm which could be applied in various fields including LIDAR and material processing^1^,^2^,^3^. The 2 μm band has been regarded as a potential transmission window for optical communications, attributed to the excellent atmospheric transmission. Fiber sources in this region are prospective to form the next-generation high-capacity optical communication networks. The networks capacity will get higher if the efficient operating spectral ranges could be extended further. Besides, fiber sources operating at longer than ~2.1 μm are also needed in other applications including nonlinear optics, medicine and remote sensing. Therefore, considering that most reported powerful Tm-doped fiber lasers (TDFLs) and amplifiers (TDFAs) were within the spectral range of 1.9–2.05 μm^4^,^5^,^6^, researchers have started to explore feasible schemes for further extension of wavelength range.

In recent two years, ultrashort-wavelength silica-based TDFLs and TDFAs have been reported^7^,^8^,^9^, which aim to fill the spectral gap between Er-doped fiber lasers/amplifiers (EDFLs/EDFAs) and TDFLs/TDFAs. However, few ultralong-wavelength silica-based TDFLs/TDFAs were reported, which is due to the gain saturation effects, large quantum defect and exponentially increase of intrinsic loss in this band. To extend the operating wavelength to >2.05 μm, researchers turned to another promising dopant Ho^3+^ and reported a powerful Ho-doped fiber laser (HDFL) which produced up to 18 W at wavelength as long as 2171 nm^10^. Besides, nonlinear schemes including Raman fiber laser and Raman fiber amplifier have been investigated to achieve ultralong wavelengths in Tm-doped fiber based laser systems. In 2014, Liu *et al*. demonstrated a 14.3 W silica-based Raman fiber amplifier seeded with a 2.4 W TDFL at 2147 nm^11^. In 2015, Jiang *et al*. reported a silica-based Raman fiber laser at >2.4 μm with the maximum output power of sub-watt when employing a pulsed 2 μm fiber laser as pump source and a piece of highly nonlinear fiber as Raman gain medium^12^, which is believed as the longest wavelength operation of silica-based fiber laser. In these reported nonlinear schemes for ultralong-wavelength laser emission, complicated structures and costly devices such as multi-stage amplifiers and powerful pulsed 2 μm fiber lasers were employed to overcome the obstacles for wavelength extension and high-power operation. Therefore, it’s in great demand to put forward a simple and inexpensive scheme such as a Tm-doped silica fiber oscillator.

Silica-based TDFLs operating at >2.1 μm with the output power of sub-watt and pumped with 0.79 μm laser diodes (LDs) have been reported previously^13^,^14^, and the maximum output powers were limited by gain saturation effects caused by strong amplified spontaneous emission (ASE). Therefore, a powerful cladding-pump source at the moderate absorption band of Tm ions would be essential to achieve the high-power laser emission of an ultralong-wavelength TDFL without ASE and parasitic oscillation effects. Considering that a moderate absorption band of Tm ions peaks at ~1.2 μm, high-power random distributed feedback fiber lasers (RDFBLs) and Raman fiber lasers operating in this spectral region are favorable choices. Both of them have been used effectively as the pump sources of mid-infrared fiber lasers. For Raman fiber laser, a 100 W-level TDFL at 1943 nm common wavelength was achieved by employing 1173 nm Raman fiber lasers as pump sources^15^. For RDFBL, a 23 W HDFL at 2050 nm was realized when pumped by a 1150 nm RDFBL, in which RDFBL could stable operate and fusion splices were believed to have little impact on the random fiber generation^16^. With the rapid progress of RDFBLs^16^,^17^,^18^,^19^,^20^, the maximum output power has reached hundred watt level^18^, which is powerful enough as a pump source for TDFLs.

In this paper, we proposed a simple and compact high-power ultralong-wavelength cladding-pumped Tm-doped silica fiber laser at 2153 nm with the output power exceeding 18 W and the slope efficiency of 25.5%. A home-made high-power RDFBL at 1173 nm^18^ was employed as pump source of TDFL. No ASE or parasitic oscillation effects were observed at the maximum output power in this scheme. We also conducted comparison experiments on the operation of 793 nm LDs-pumped TDFLs at 2153 nm.

## Results

**Figure 1:**
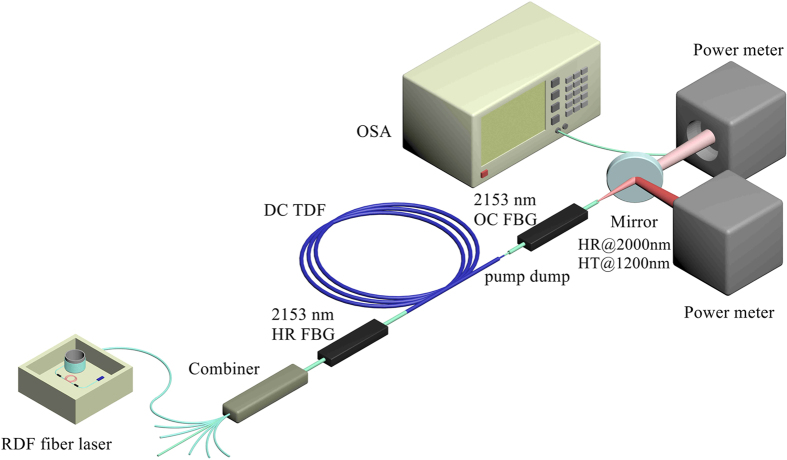
Experimental setup of 2153 nm TDFL cladding-pumped with a random distributed feedback fiber laser. RDF fiber laser: random distributed feedback fiber laser; FBG: fiber Bragg grating; HR: high-reflectivity; OC: output coupler; DC TDF: double-cladding Tm-doped fiber; Mirror: dichroic mirror; OSA: optical spectrum analyzer.

The experimental setup of high-power TDFL at 2153 nm was shown in Fig. 1. A home-made short-cavity RDFBL served as pump source of TDFL. The output power of RDFBL was launched into the cavity of TDFL from one pump port of a (6 + 1) × 1 pump-signal combiner by fusion splicing directly. The pigtail of RDFBL was SMF-28 with the core/cladding diameter of 8.2/125 μm. The pump ports and signal port of pump-signal combiner were fibers with the core/cladding diameter of 105/125 μm and 10/125 μm, respectively. The output port of combiner was the fiber with core/cladding diameter of 25/250 μm, and core/cladding numerical aperture (NA) of 0.11/0.46. All the vacant signal port and pump ports were angle-cleaved to prevent unwanted feedback. A pair of FBGs was employed to form laser cavity. The high-reflectivity (HR) FBG has a center wavelength of 2153.2 nm, a spectral bandwidth of 1.3 nm and a reflectivity of 99.9%. The reflectivity of the output coupler (OC) FBG was 58.9%, with the center wavelength of 2153.2 nm and bandwidth of 1.0 nm. The passive fiber used in FBGs matched well with the output port of combiner, with the same fiber geometry and NA. According to the cladding absorption coefficient of ~2.0 dB/m at 1173 nm, we chose double-cladding Tm-doped fiber (DC TDF) with the length of 7.2 m to ensure sufficient absorption of pump light. Considering that there were still unabsorbed pump light in the cavity, we stripped pump light by coating the fusion splice between DC TDF and OC FBG with high refractive index gel. The output port of OC FBG was angle-cleaved to avoid feedback from fiber facet. The TDFL including all the fusion splices was fixed on a water-cooled heat sink. We also used a dichroic mirror with high reflectivity at ~2.0 μm and high transmittance at ~1.2 μm to split 2153 nm signal laser and residual pump laser. The mirror was carefully adjusted and fixed to guarantee the maximum power of reflected signal laser obtained.

**Figure 2:**
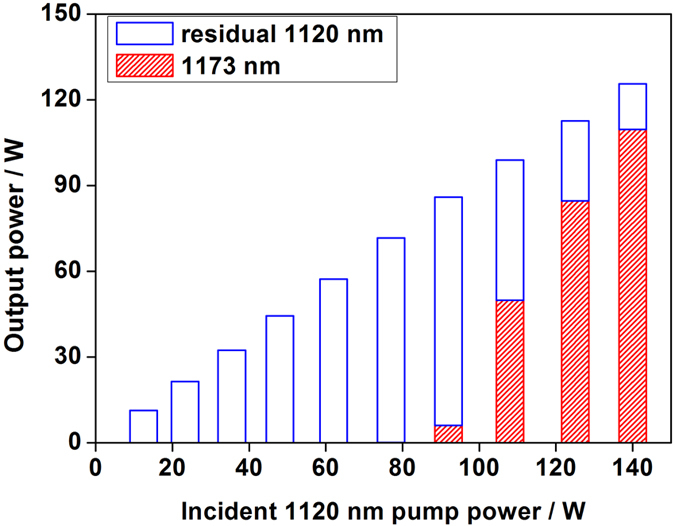
Output power and spectral composition of random distributed feedback fiber laser.

We measured the output power and spectral composition of RDFBL which served as pump source of TDFL, and the results was shown in Fig. 2. The output power of RDFBL reached 125.7 W, including 109.7 W signal power at 1173 nm and 16 W residual pump power at 1120 nm, when launched 1120 nm Yb-doped fiber laser with the power of 140 W into RDFBL. The threshold pump power of RDFBL was ~80 W, and the optical-to-optical efficiency was ~77% when launched 140 W 1120 nm pump laser. The lasers emitted from RDFBL were directly launched into TDFL without stripping residual 1120 nm fiber laser.

**Figure 3:**
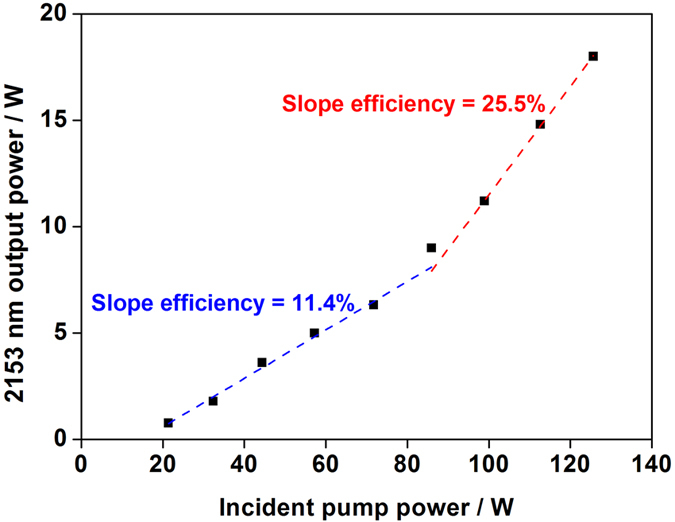
Output power of TDFL versus the total incident pump power.

Then we measured the output power of 2153 nm TDFL. The laser reflected by dichroic mirror was regarded as the expected signal laser, which successfully stripped the residual pump laser emitting from the angle-cleaved output port of OC FBG. As shown in Fig. 3, the maximum power of reflected 2153 nm fiber laser reached 18 W, when the total pump power was 125.7 W (87.3% @1173 nm, 12.7% @1120 nm). It is worth noting that the slope efficiency of TDFL changed with the composition of pump laser. When the incident pump power was less than ~75 W and purely centered at 1120 nm, the slope efficiency of TDFL was only 11.4%. Afterwards, the slope efficiency rapidly increased to 25.5% when the incident pump power exceeding ~75 W. Since the ~75 W incident pump power of TDFL corresponded to the output power at the threshold of RDFBL, it is indicated that the threshold of this short-cavity RDFBL was not obviously changed after employed as pump source of TDFL. Thus, the laser performances of RDFBL could be kept in the experiment if we had carefully managed parasitic reflections. The differences of efficiencies when pumped at 1120 nm and 1173 nm should be attributed to the various excited-state absorption (ESA) intensities which could resulted in different quantum efficiencies. It’s estimated that the quantum efficiency when pumped with 1173 nm pump laser was at least 56%. And the quantum efficiency with the pump source at 1120 nm in the experiment was 23%, which is rather lower than that of 1173 nm. In the ESA process of TDFL, Tm-doped silica fiber emitted up-conversion fluorescence at ~480 nm and ~810 nm^15^,^21^. Bright blue fluorescence (~480 nm) was observed emitting from the active fiber in the experiment, when 1120 nm pump laser started to pour into the TDFL. Due to the quantum defect and ESA effect when pumped with RDFBL, thermal load accumulating in Tm-doped fiber became a challenge for long-wavelength TDFL in this scheme. To ensure the safety of fiber system, the temperature of polymer coating should be kept below 80 °C, which limited the maximum output power. Further improving the efficiency of TDFL and restraining the ESA process could help to ease the thermal load. Thus, by optimizing the length of Tm-doped fiber laser, reflectivity of FBG, increasing the efficiency of RDFBL and employing better temperature management, the maximum output power of long-wavelength TDFL could be further enhanced.

**Figure 4:**
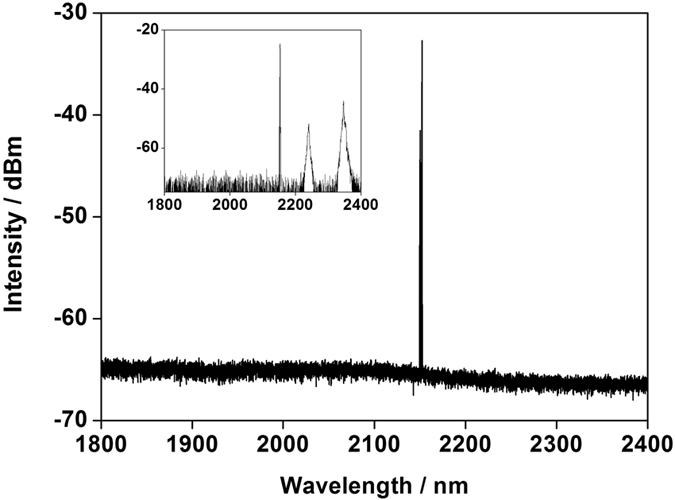
Optical spectrum of fiber laser reflected by a dichroic mirror. Inset: Optical spectrum of transmitted laser.

Actually, the moderate absorption cross section of Tm-doped silica fiber at 1173 nm was the key to achieve high-power laser emission at long-wavelength. The spectrum of reflected and transmitted laser with the maximum output power was demonstrated in Fig. 4 and its inset, respectively. The laser reflected by dichroic mirror was pure which centered at 2153 nm and the 3 dB bandwidth was ~0.9 nm, and there was no ASE or parasitic oscillation in the range of 1900–2000 nm where the gain was strong. The transmitted laser, which was depicted in the inset of Fig. 4, consisted of signal laser at 2153 nm, the second-order diffraction light of 1120 nm and 1173 nm pump laser. There is also no ASE or parasitic oscillation observed in the transmitted spectrum. Therefore, it’s indicated that employing powerful 1173 nm random distributed feedback fiber laser is a feasible and promising pump scheme for high-power ultralong-wavelength TDFL.

**Figure 5:**
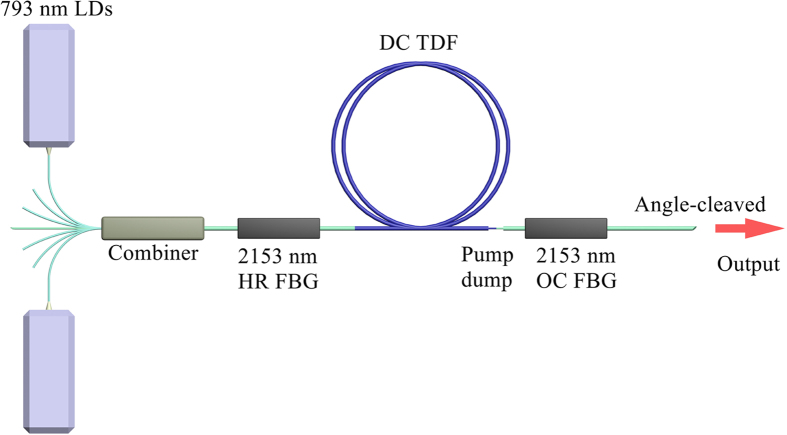
Experimental setup of a 2153 nm TDFL pumped with 793 nm LDs. LD: laser diode; FBG: fiber Bragg grating; HR: high-reflectivity; OC: output coupler; DC TDF: double-cladding Tm-doped fiber.

We also compared the results of 2153 nm Tm-doped fiber laser when pumped with high-power 793 nm LDs. The experimental setup of LDs-pumped 2153 nm TDFL was demonstrated in Fig. 5. Two commercial 793 nm LDs with the maximum output power exceeding 150 W were employed as pump sources, and the pump lasers were launched into TDFL via a (6 + 1) × 1 signal-pump combiner. A pair of FBGs which were the same with those mentioned above formed the laser cavity. All the vacant pigtails were angle-cleaved to avoid feedback from fiber facets. The cladding absorption coefficient of this DC TDF was ~8.5 dB/m when cladding-pumped with 793 nm lasers. Therefore, to make fair comparison with RDFBL pumping scheme, 2.7 m TDF was chosen firstly to ensure sufficient and similar pump absorption. Then we replaced it with a length of 3.6 m TDF and investigate how much the threshold of parasitic oscillation increased by.

**Figure 6:**
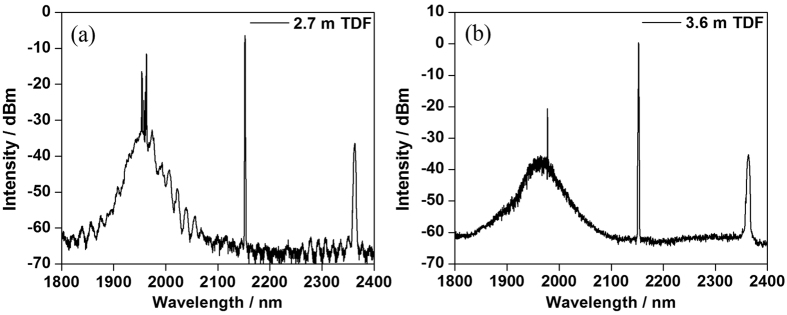
Optical spectra of TDFLs with the output power of ~2 W when pumped with LDs. (**a**) Optical spectrum of TDFL with 2.7 m TDF; (**b**) Optical spectrum of TDFL with 3.6 m TDF.

The maximum output powers of LDs-pumped TDFLs with various active fiber lengths were all limited to ~2 W by the strong ASE and parasitic oscillation in the range of 1900–2000 nm. In the experiment, when the length of TDF increased by 0.9 m, only 0.2 W was increased for the threshold of parasitic oscillation. We didn’t further enhance the length of active fiber in 793 nm LD pumping scheme, because it would introduce increased loss of signal power due to reabsorption process and the parasitic oscillation threshold was not dramatically increased in previous experiment. The optical spectra of LDs-pumped TDFLs with the output power of ~2 W were shown in Fig. 6. Obvious ASE and parasitic oscillation effects have been observed, which confined the improvement of signal laser at 2153 nm. Due to the strong absorption processes within the range of unexpected wavelength ranges, it’s concluded that using LDs with central wavelength at 793 nm is not an eligible scheme for ultralong-wavelength TDFLs.

**Figure 7:**
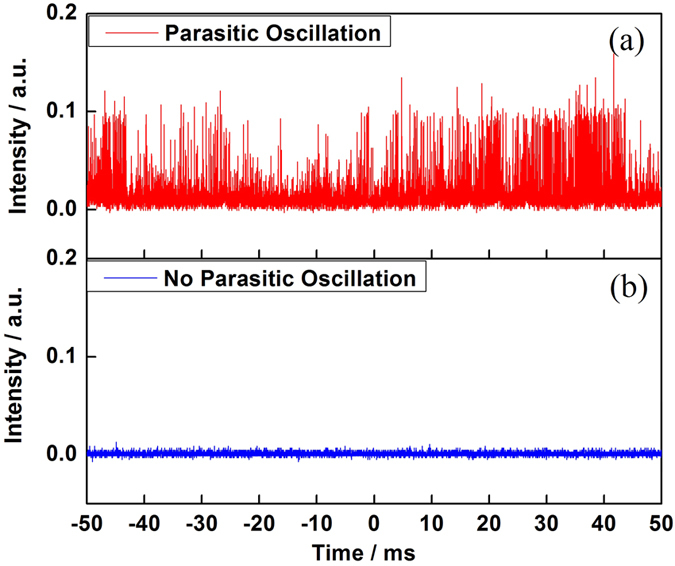
Comparison of temporal characteristics with different output power. (**a**) Parasitic oscillation observed; (**b**) No parasitic oscillation.

The parasitic oscillation occurred in the TDFLs not only confined the power scalability, but also had side-effects on temporal characteristics. As shown in Fig. 7, we compared the temporal characteristics of TDFLs after (a) and before (b) the parasitic oscillation occurred. After the threshold of parasitic oscillation reached, the temporal characteristics became highly unstable, which was harmful to optical devices and endanger the steady operation of laser system.

In conclusion, we demonstrated a high-power ultralong-wavelength TDFL operating at 2153 nm with the maximum output power exceeding 18 W and a slope efficiency of 25.5%. A short-cavity RDFBL with central wavelength of 1173 nm was employed as pump source of this TDFL. The laser performances of RDFBL were not obviously influenced after spliced with the pump port of TDFL. Power scaling of TDFL in this experiment is confined by the thermal endurance of polymer coating, which could be further improved by optimizing the length of active fiber, reflectivity of FBG, increasing efficiency of RDFBL and using better temperature management. Comparison experiments were conducted to verify the feasibility of RDFBL pumping scheme. The maximum output powers of 2153 nm TDFLs pumped with 793 nm LDs were at the level of ~2 W, which were limited by strong ASE and parasitic oscillation effects. And there was 0.2 W increased for the threshold of parasitic oscillation, when we altered the length of TDF from 2.7 m to 3.6 m in the LDs-pumped TDFLs. The unfavorable phenomenon, unstable temporal characteristics, was also observed in the experiments. Comparing these two pump schemes, the maximum output power improved by 8 times from ~2 W to ~18 W, which is quite remarkable. Hence, employing powerful RDFBL at 1173 nm is regarded as a feasible pump scheme of high-power ultralong-wavelength TDFLs, which is quite promising to meet the requirements of tremendous applications.

## Discussion

To demonstrate the importance of the proposed system in this paper, we compared our results and efficiency to other fiber sources in this wavelength band. As stated previously, the maximum output power and slope efficiency of this RDFBL pumped 2153 nm TDFL are 18 W and 25.5%, respectively. For Raman fiber amplifier^11^, the maximum power was 14.3 W at 2147 nm, and the conversion efficiency was 38.5% from 1963 nm to 2147 nm in 50 m highly nonlinear fiber which acts as the Raman gain medium. For long-wavelength TDFL directly pumped with 0.79 μm LDs^14^, the maximum output power reached ~1 W at 2151 m when 8 W pump light was launched into the cavity, and the slope efficiency was 30%. For HDFL in this wavelength region^10^, the maximum output power reached ~23 W at 2.15 μm, and the slope efficiency was ~33% with respect to launched pump power. In the HDFL system, free-optics devices including lens, diffraction grating and dichroic mirrors were employed. 793 nm LDs and Tm-doped fiber were used to construct the pump TDFL, and special HDF with an internal fluorine-doped layer was employed as gain fiber. Compared to this, all-fiber configuration could be easily achieved in the proposed TDFL system by using common active fiber and pump combiner. 976 nm LDs, Yb-doped fiber and passive fiber were employed to build pump RDFBL, and conventional Tm-doped fiber was used as gain fiber. All the devices used in the TDFL, including pump source and gain fiber, are more mature and cheaper than those in the reported HDFL. Therefore, this all-fiber TDFL with comparable output power and efficiency is a competitive scheme to achieve high power emission at the wavelength longer than 2.15 μm. The mature devices, cheap pump source and all-fiber configuration make this TDFL cost effective and compact, which is very important for various applications.

Besides, RDFBL is not the only choice of pump laser to achieve high-power laser emission from ultralong-wavelength TDFL, other powerful fiber sources such as Raman fiber laser operating within this moderate absorption band of Tm ions could also be used as pump laser effectively. To clarify whether some differences exist on the slope efficiency, power threshold and output spectrum of TDFL pumped by RDFBL and Raman fiber laser, we did comparsion experiments and the laser system configurations were simplified as following: A RDFBL and a Raman fiber laser with 500 m-long SMF-28 fiber operating at 1173 nm were used as pump source, respectively. And the TDFL consisted of a pair of 2050 nm FBGs, a piece of 6.6 m TDF with the cladding absorption coefficient of ~2.0 dB/m at 1173 nm. Other parts were the same as the laser configuration in Fig. 1. The slope efficiencies were 34.8% and 34.1% for RDFBL- and Raman fiber laser-pumped TDFL respectively, which may be induced from measurement error and could be neglected at this power level. We didn’t observe any obvious differences on power thresholds and output spectra based on these two pumping scheme. With the same length of SMF-28 fiber in RDFBL and Raman fiber laser, their power scalabilities were different with the 2^nd^ order Stokes thresholds of ~45 W and ~25 W, due to different feedbacks. Thus, the output power of RDFBL at 1173 nm could get higher compared to that of Raman fiber laser. However, for such ten-watt level ultralong-wavelength TDFL proposed in this paper, there is no priority for pump lasers with hundred-watt output power for the time being.

It is also worth noting that the final efficiency of this silica fiber based system is affected by infrared (IR) absorption in silica due to the ultralong wavelength operation. To demonstrate this, we estimate the loss induced by silica IR absorption in our system. As far as we know, silica IR absorption exponentially increases with the wavelength, and reaches ~0.1 dB/m at 2153 nm, the operating wavelength of current TDFL. As the total length of TDFL is less than 8.5 m including the fiber length of FBG pigtails, 18% of signal intensity was absorbed by silica fiber, which leads to a ~5.5% decrease of final efficiency.

## Method

### Measurement Method

An optical spectrum analyzer (OSA) with a resolution of 0.02 nm and the scanning spectral range from 1600 nm to 2400 nm was employed to measure the spectrum of both reflected and transmitted lasers. Two power meters were used to measure the output power of reflected and transmitted laser. The pigtails of TDFL, dichroic mirror and two power meters were fixed very close to each other. Thus, the output lasers could be measured directly without collimating, due to the large target surface of power meters. When measuring spectra, a piece of single-mode fiber was used. We fixed the fiber opposite to the directions of reflected and transmitted beams respectively to couple those beams into OSA. During the experiments, the signal-to-noise ratios (SNRs) of both spectra were guaranteed to be higher than ~30 dB. Temporal characteristics of TDFLs are detected by employing a 1 GHz oscilloscope and a photodetector with the rise time of 25 ns.

## Additional Information

**How to cite this article**: Jin, X. *et al*. High-power ultralong-wavelength Tm-doped silica fiber laser cladding-pumped with a random distributed feedback fiber laser. *Sci. Rep*. **6**, 30052; doi: 10.1038/srep30052 (2016).
